# Anatomical investigation of the pelvic urogenital fascia in 10 formalin-fixed female cadavers: novel insights into the laparoscopic total mesometrial resection

**DOI:** 10.1186/s12893-023-02239-5

**Published:** 2023-10-27

**Authors:** Zheqi Zhou, Bin Li, Jinsong Zhou, Yanbing Ma, Yang Zhao, Cong Tong, Hui Wang, Dian Jin, Yujie Li, Likun Yan

**Affiliations:** 1 Department of General Surgery, Shaanxi Provincial People’s Hospital, Xi’an, 710068 China; 2 Department of Obstetrics and Gynecology, Shaanxi Provincial People’s Hospital, Xi’an, 710068 China; 3 Yan’an University, Yan’an, 716000 China; 4 Department of Human Anatomy, Histology and Embryology, School of Basic Medical Sciences, Xi’an Jiaotong University Health Science Center, Xi’an, Shaanxi 710061 China; 5 Editorial Board of Shaanxi Popular Medical Education, Xi’an, 710068 China

**Keywords:** Urogenital fascia, Total mesometrial resection, Extraperitoneal space, Broad ligament, Round ligament, Pelvic fascial structures

## Abstract

**Purpose:**

Previous anatomical studies of the urogenital fascia (UGF) have focused on males, and there is a lack of relevant anatomical studies on the distribution of the extraperitoneal UGF in females.

**Methods:**

In this investigation, guided by the embryonic development of the female urogenital system, the ventral pelvic fascia structure of 10 female cadavers was dissected, and the distribution and morphology of female extraperitoneal UGF were observed, recorded in text, photographs and video, and 3D modeling was performed.

**Results:**

We find that in the female extraperitoneal space there is a migratory fascial structure, the UGF, which surrounds the urogenital system and extends from the perinephric region to the pelvis along with the development of the urogenital organs. The two layers of the UGF are composed of loose connective tissue rich in fat that surrounds the urogenital organs, their accessory vascular structures, and the nerves of the abdominopelvic cavity. In the pelvis, it participates in the formation of the ligamentous structures around the rectum and uterus. Finally, it surrounds the bladder and gradually moves into the loose connective tissue of the medial umbilical fold.

**Conclusions:**

Sorting out the distribution characteristics of UGF has some reference value for studying the metastasis of gynecological tumors, the biomechanical structure of the female pelvis, and the surgical methods of gynecology, colorectal surgery, and hernia surgery.

**Supplementary Information:**

The online version contains supplementary material available at 10.1186/s12893-023-02239-5.

## Introduction

The combination of embryology, anatomy, and surgery has helped surgeons identify anatomical problems, analyze anatomical structures, and innovate surgical methods. With the widespread development of minimally invasive surgery and pursuit of safe and efficient operation plans, surgical membrane anatomy has made great progress. Surgeons from different specialties, such as colorectal surgery, obstetrics and gynecology, and urology, mark and name the fascial structures based on the characteristics of their respective specialties by separating their respective surgical areas, and this naming based on surgical practice lacks standardization and uniformity [1–3]. This confuses the naming of pelvic floor fascial structures, which can easily lead to misunderstanding [4]. The female pelvis is a complex anatomical region that includes pelvic organs, muscles, nerves, blood vessels, and fascia, and the pelvic floor fascia is continuous and nested [5, 6]. The anatomy of these pelvic fascial structures is currently poorly described and often misunderstood [7]. Through previous anatomical studies of 10 formalin-fixed male cadavers, the author’s team discovered the existence of an intact and continuous extraperitoneal fascial system surrounding the abdominal and pelvic cavities, located in the extraperitoneal space, originating from the renal fascia, crossing the pelvis posteriorly and extending into the preperitoneal space as a continuation of the fascial structure of the bladder, and entering the inguinal canal as a continuation of the internal spermatic fascia [8–11]. The author’s team through the embryonic development of human urogenital organs as a guide combined with the anatomical findings named it the urogenital fascia (UGF), which is similar to the localized findings of Diarra [12] et al. but we made systematic additions and refinements. The distribution of the UGF in the female pelvis and its adjacency to the pelvic organs is currently unclear. To explore the distribution and adjacency of UGF and other fascial structures in the female retroperitoneum and pelvis, the author’s team dissected the abdominal and pelvic fascial structures of 10 female cadavers in formalin with a holistic view of the embryonic development of the female genitourinary system as a guideline to explore the value of the application of UGF in laparoscopic total mesometrial resection.

## Materials and methods

### Materials

Ten formalin-treated female cadavers provided by the Department of Human Anatomy and Histoembryology, Health Science Center, Xi’an Jiaotong University were dissected using a set of dissection equipment and a camera. The cadavers used in this study had intact retroperitoneal and pelvic cavities, the type of study was anatomical and surgical, and the cadavers were exposed only to formalin solution and no other fluids during the entire study. There was no pathological evaluation of cadaveric specimens in this investigation, and the entire study was conducted by two professors of human anatomy, two professors of general surgery, one professor of obstetrics and gynecology, and three postgraduate students, who were systematically trained and qualified for anatomical studies. The entire study was conducted in strict accordance with the protocol approved by the Biomedical Ethics Committee of Xi’an Jiaotong University (Ethics License No. 2014 − 0303). For educational and scientific research purposes, written informed consent was obtained from the immediate family members of the deceased. The format of the informed consent form followed the guidelines of the China Organ Donation Management Center. The methods of this study follow the CACTUS guidelines, which provide guidance for anatomical studies and contribute to the quality of research methods [13].

### Methods

The peritoneum was incised longitudinally at the bilateral paracolic gutters, the colon was lifted to expose the retroperitoneal space, and the perirenal fascia was carefully dissected to observe its continuation into the pelvis. The specimen was dissected with a saw along the highest point of the iliac crest and the sagittal plane of the pelvis, respectively, and the UGF, internal iliac artery, pelvic splanchnic nerves, and pelvic plexus were carefully dissected, and the rectum, uterus, broad ligament, round ligament of uterus, upper vagina, fascial structures around the bladder, and preperitoneal fascia were dissected. The peritoneum of the hemipelvic specimen was completely and carefully peeled off, fully exposing the underlying UGF. Finally, the aforementioned fascial structures and the organs, blood vessels, and nerves within them were dissected from the cadaveric specimen along with the iliac blood vessels and flattened, taking care to preserve their integrity. Finally, their morphology was observed and documented through text, photographs, 3D modeling, and video.

## Results

**Fig. 1:**
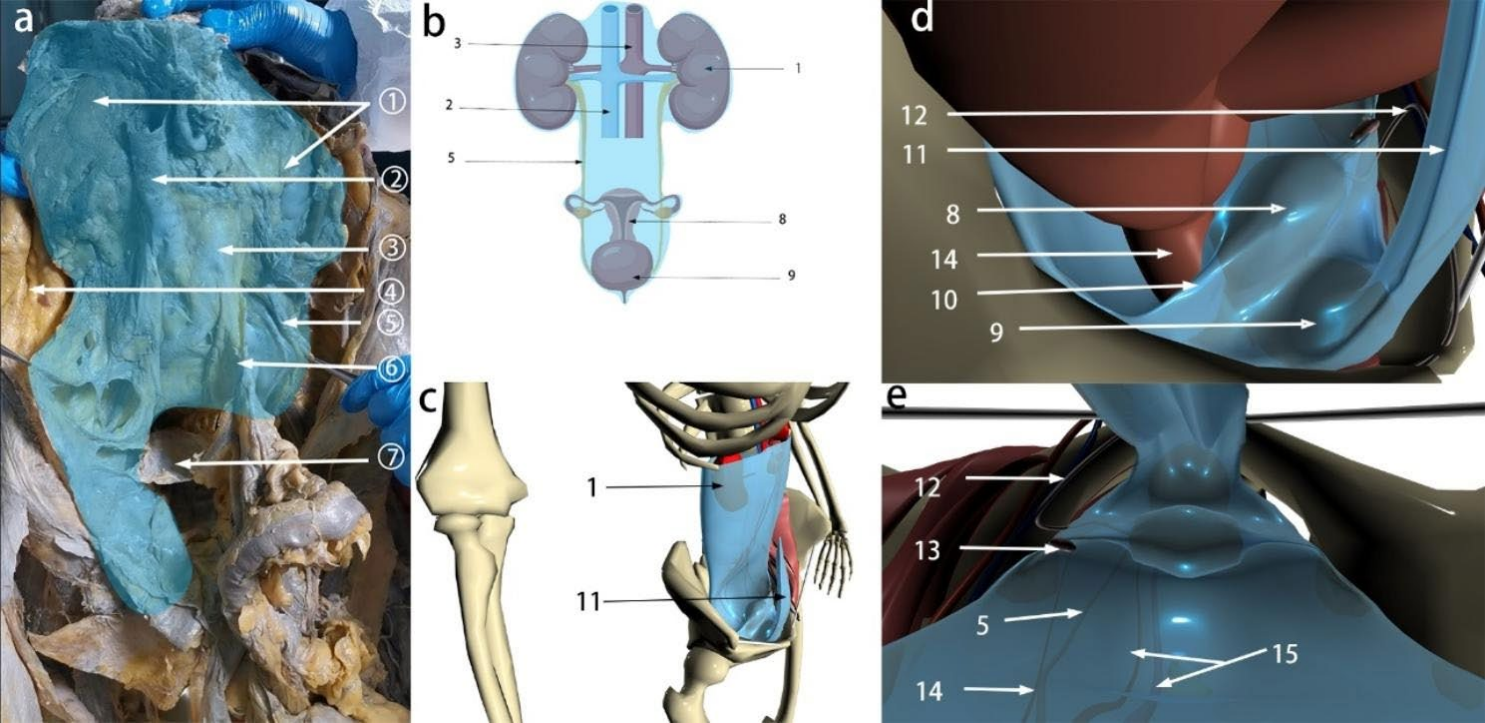
The UGF overall view and modeling. (**a**) Cadaveric anatomical view of the UGF; (**b**) The UGF pattern view; (**c**-**e**) 3D modeling view. (1) Kidney; (2) Inferior vena cava; (3) Abdominal aorta; (4) Transversalis fascia; (5) Ureter; (6) UGF migration into the mesentery; (7) Peritoneum; (8) Uterus; (9) Bladder; (10) Broad ligament; 11. Medial umbilical fold; 12. The round uterine ligament(left); 13. Ovary (left); 14. Rectum; 15. Internal iliac vessel (left). The blue membranous structure in the 3D modeling Figures is the UGF. The blue shading in the (**a**-**b**) Figures is UGF

**Fig. 2:**
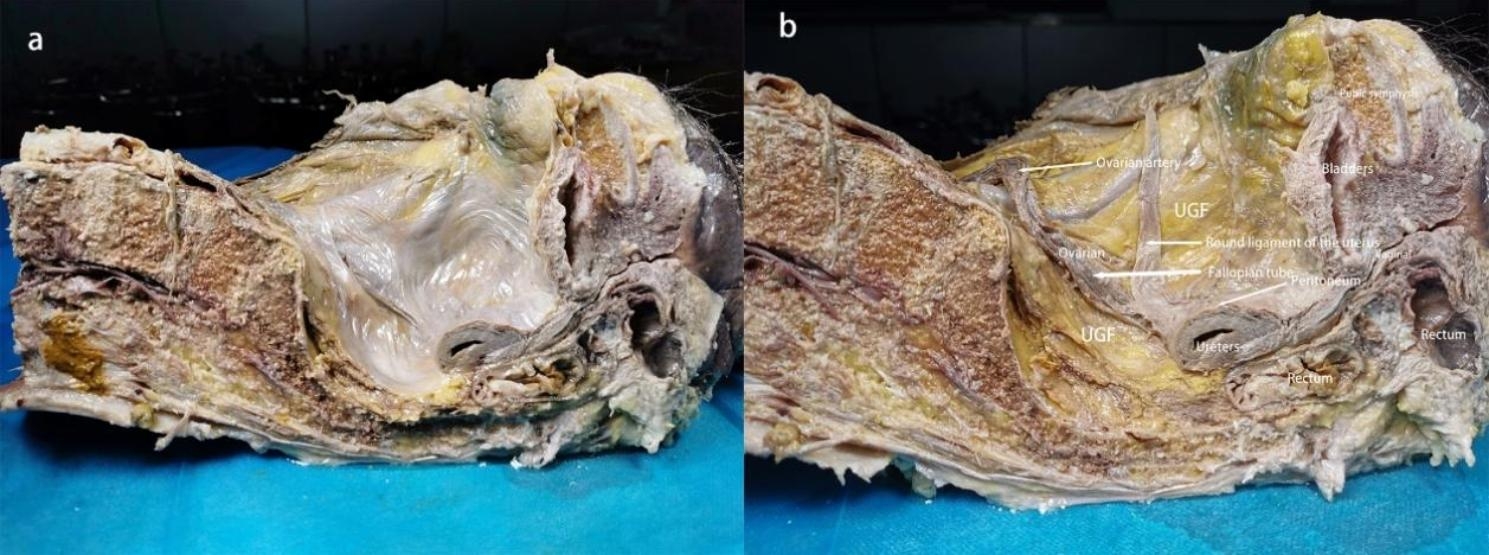
Demonstration of hemipelvic UGF. (**a**) Preservation of peritoneum; (**b**) Removal of peritoneum

**Fig. 3:**
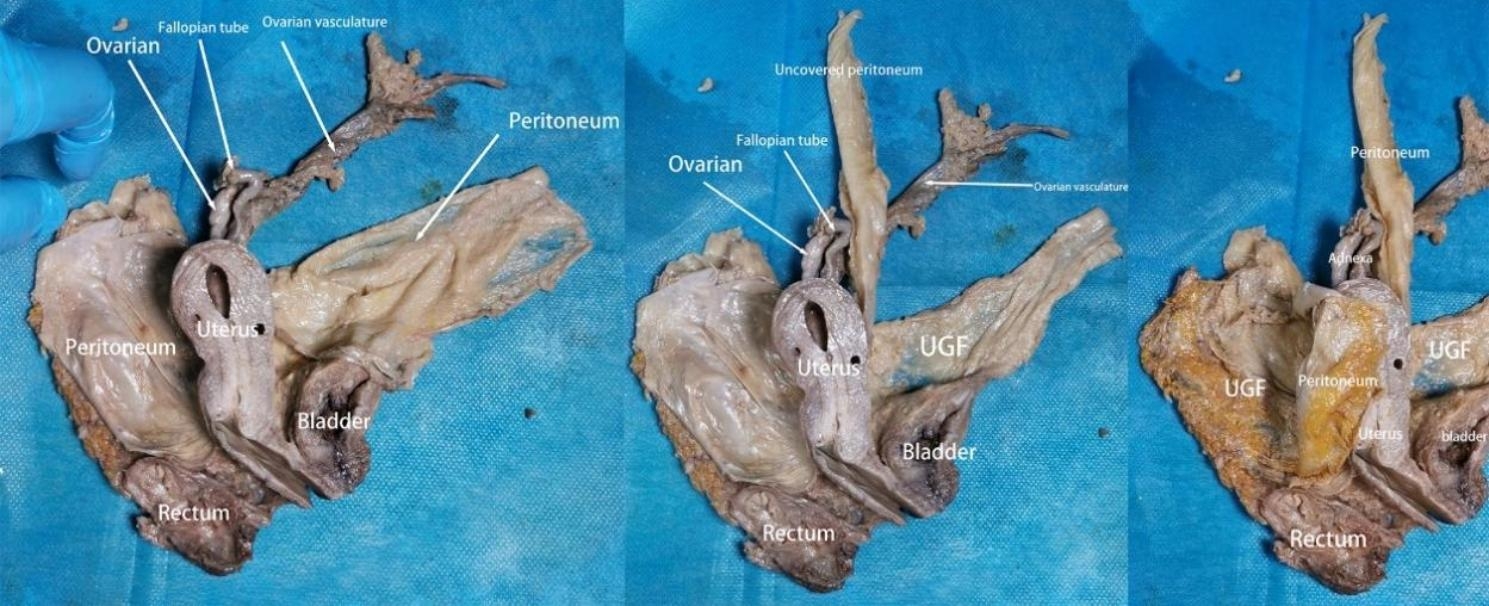
Semi-pelvic position UGF removal view

**Fig. 4:**
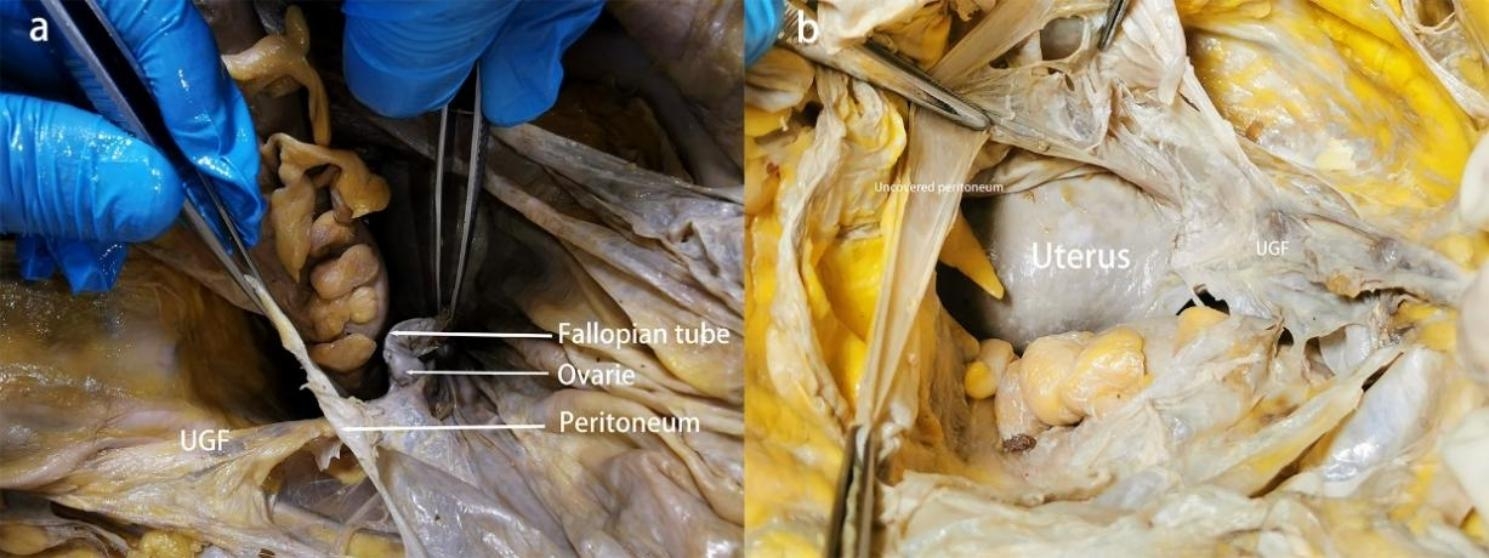
The UGF migrates into the parietal uterus

**Fig. 5:**
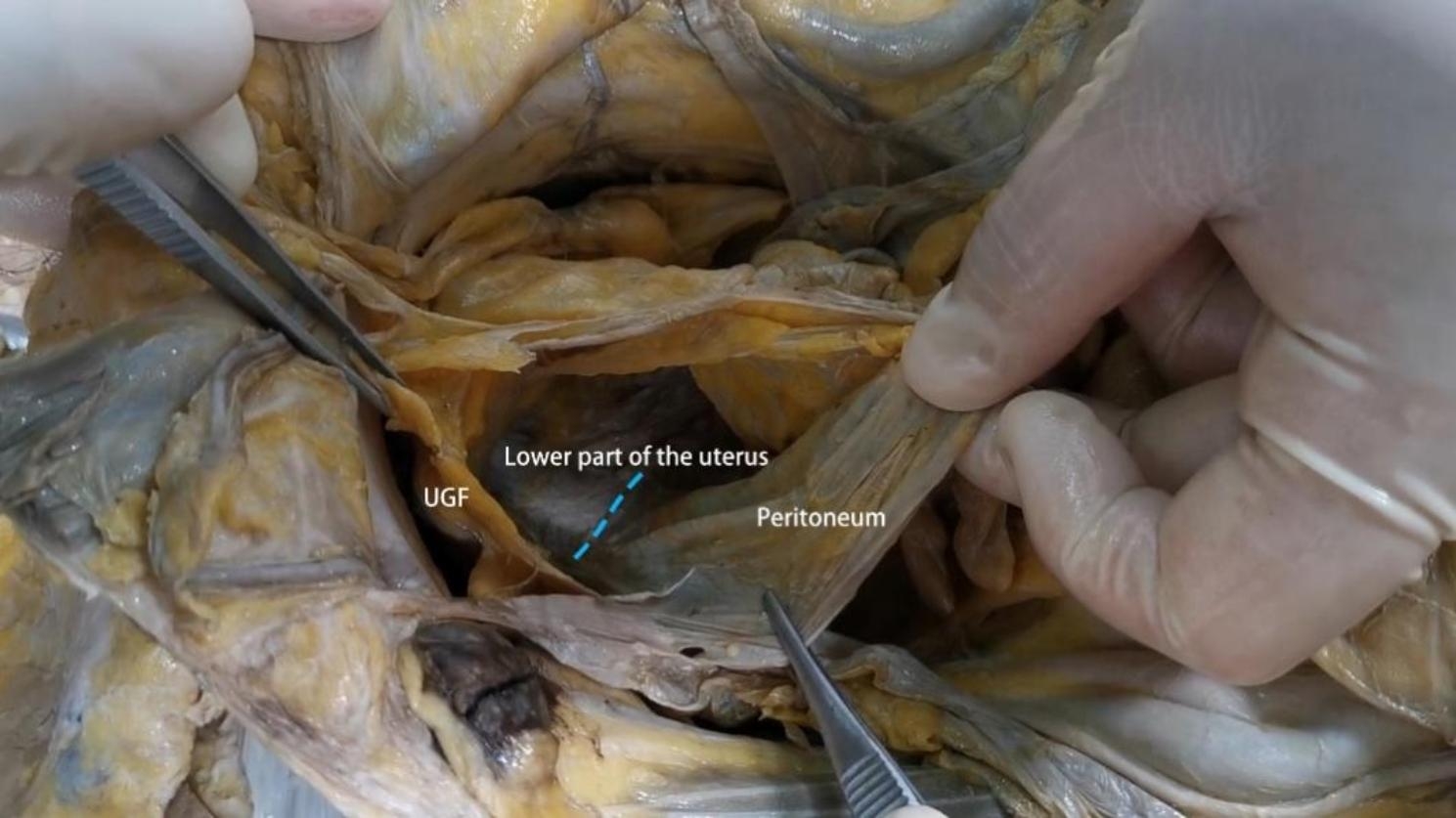
Sparse distribution of UGF on the surface of the uterus and difficulty in separating it from the peritoneum

**Fig. 6:**
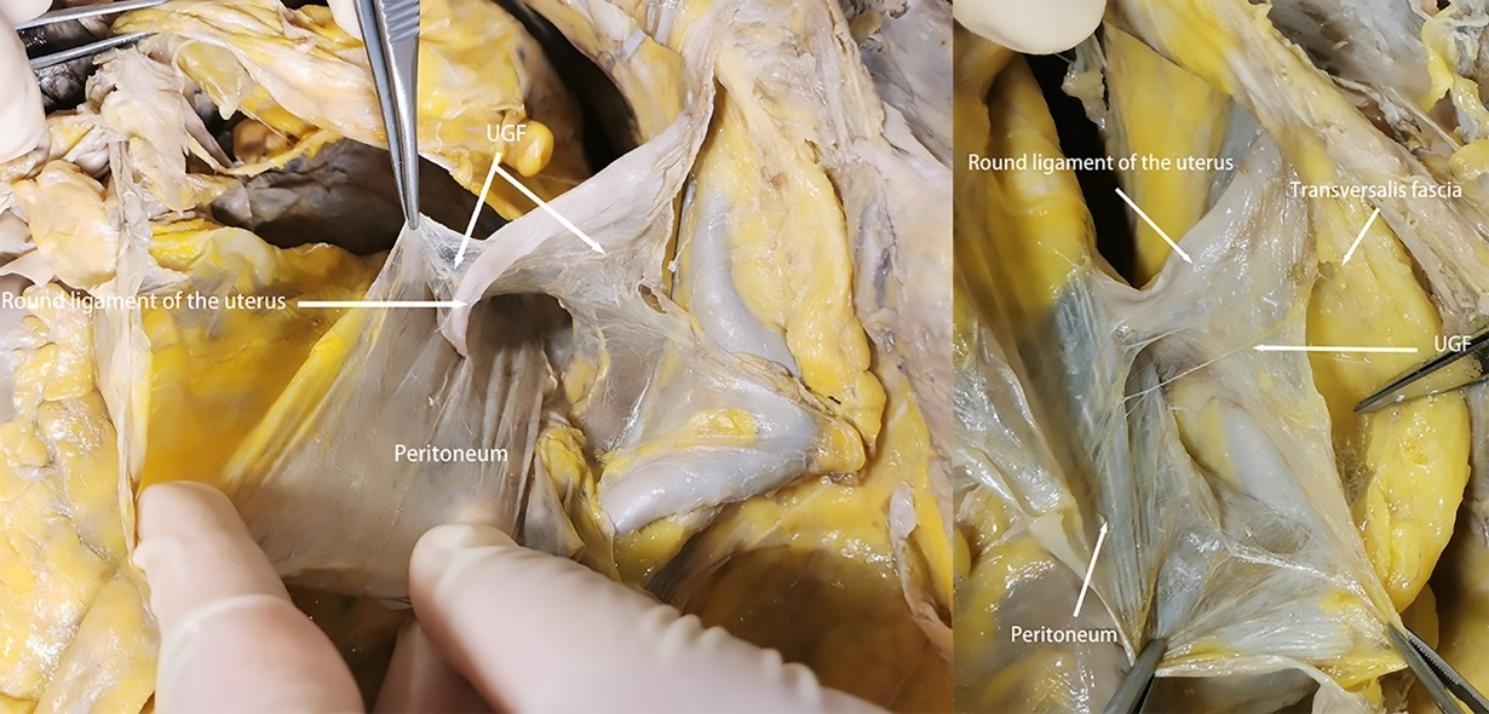
Relationship between the round uterine ligament and UGF

Our research has shown that outside of the peritoneum there is an intact migratory fascial structure that surrounds the urogenital system known as the UGF. The fascia extends anteriorly and posteriorly around and into the female pelvis with the development of the genitourinary organs. The two layers of the UGF are loose connective tissue rich in fat that surrounds the kidneys, ureters, fallopian tubes, ovaries, hypogastric nerves, uterus, bladder, and their accessory vascular structures. The side near the center of the body is called the visceral layer, and the other side is called the mural layer. At the upper pole of the kidney, the visceral and mural layers of the UGF surround the kidney, and they originate from the intermediate mesoderm like the kidney, and we can consider the anterior and posterior renal fascia as the starting segments of the visceral and mural layers of the UGF. In the midline, the visceral and mural layers of the UGF fuse in front of the aorta and inferior vena cava. Laterally, the UGF surrounds the ovarian vessels and is externally bordered by the laterocostal fascia. At the inferior pole of the kidney, the UGF continues to expand and encompasses the genitourinary vessels, ureters, and submental nerves as it migrates toward the pelvis (Fig. 1). Its visceral layer, in the process of continuing anteriorly and downwardly along the lateral wall of the pelvis to the 4th sacral vertebra, forms Waldeyer’s fascia between the fascia propria of the rectum and the sacrum, and continues anteriorly as the prehypogastric nerve fascia, the lateral ligament of the rectum, the uterosacral ligament, the round ligament of the uterus, the ovarian mesentery, and the suspensory ligament of the ovary, the loose connective tissue in the broad ligament, and then encapsulates the cervix with part of the upper part of the vagina, It then encircles the cervix, with part of the upper vagina gradually migrating as the loose connective tissue at the base of the bladder and finally moving upward along the abdominal wall to the loose connective tissue in the medial umbilical fold (Figs. 2, 3 and 4). In the posterior vaginal wall, the visceral layer of the UGF continues to the pelvic floor, and in the part of the uterus above the cervix, the UGF is very thin and difficult to separate from the peritoneum, which is an anatomical feature here. Between the anterior vaginal wall and the posterior bladder wall, the visceral layer of the UGF continues posteriorly from the posterior bladder wall to the cervix, and above the cervix, the UGF is also very thin and difficult to separate from the peritoneum (Fig. 5). The round ligament of the uterus within the broad ligament is surrounded by the UGF, which extends outward from the inner ring, where the UGF is also very thin or absent (Fig. 6). The mural layer, on the other hand, extends inferiorly from the posterior renal fascia to the presacral and pelvic floor fasciae before merging with the visceral layer to form the medial umbilical fold.

.

## Discussion

Embryology helps us to understand the continuity of membrane anatomy. The theories of mesenteric anatomy, fascial anatomy, and membrane anatomy reveal the structure and function of membranes from different perspectives [14, 15]. Fascia is a widely used but ill-defined anatomical term that has been defined differently in different studies. The 2016 Fascial Nomenclature Committee (FNC) finally defined the fascial system as a three-dimensional continuum of soft, collagenous, loose and dense fibrous connective tissue that runs through the body and includes connective tissue structures such as adipose tissue, neurovascular sheaths, deep and superficial fascia, and visceral fascia [16]. Fascial structures undergo positional changes during human growth and development. Urogenital organs from the intermediate mesoderm-cloaca as extraperitoneal organs surrounded by a fascial system different from the peritoneal structure. Numerous scholars have studied it to some extent through anatomical investigation, but the understanding is not yet unified. The addition and development of the concept of UGF is inextricably linked to recent studies related to inguinal hernia, where early anatomical studies of the UGF were more limited and often stopped at the retroperitoneum. In 1997, Stoppa and Diarra observed the phenomenon that the vas deferens and spermatic vessels were surrounded by the fascial structure in the inguinal canal and named this fascial structure as the spermatic sheath and considered the spermatic sheath as an extension of the retroperitoneal fascial structures, and in the same year, Diarra followed with the idea about the migration of the UGF towards the pelvis and explained the naming of the UGF about the embryonic origin of the urogenital system [7, 12]. Later, both Yang and Mirilas referred to the continuation of the renal fascia into the pelvis by studying the distribution of the extraperitoneal space and the continuity of the renal fascia with the prehypogastric nerve fascia, respectively [17, 18]. In 2017, the author’s team complemented the concept of UGF with anatomical studies and proposed for the first time that UGF is a unified fascial structure that exists in the retroperitoneum, pelvis, and with the preperitoneum. However, research on the UGF has focused on males, and there are few anatomical studies of the female UGF. The results of this investigation complement the anatomical distribution of female pelvic UGF and its comparative relationship with other fasciae, and improve the research system of UGF.

How the UGF migrates from the retroperitoneum to the pelvis can be understood from the embryonic development of the female genitourinary system. At 4–5 weeks of embryonic development, the mesonephric ducts formed from the intermediate mesoderm extend caudally into the cloaca. Immediately thereafter, the ureteric buds emerge from the end of the mesonephric duct and return to the intermediate mesoderm to induce differentiation into the kidney [19, 20]. This process can be understood as a migration of the intermediate mesoderm into the cloaca and a return migration of the ureteric buds into the intermediate mesoderm. The UGF, as a fascial structure surrounding the genitourinary organs, consequently extends between the retroperitoneum and the pelvis. Hayes [21] proposed that there are three types of retroperitoneal fasciae: migrating fasciae, fusion fasciae, and parietal fasciae, in which migrating fasciae arise because the continuous migration and growth of the organ during embryonic development gradually exerts pressure on the loose connective tissue surrounding the organ, causing the fibers in the loose connective tissue to develop a linear orientation and eventually compress into a fascia-like structure. This observation is consistent with the author’s findings of UGF distribution in the extraperitoneal space by dissection. Fascial structures, like the peritoneum, are derived from embryonic mesoderm, but the difference is that the peritoneum is derived from a membrane-like structure formed by mesothelialization during the formation of the embryonic body cavity, whereas the fascia is composed of connective tissues derived from the mesoderm. Combined with the relative migration between membrane structures during embryonic development, resulting in an integral separable sacral plane between the UGF and the peritoneum in the abdominopelvic cavity. Conventional anatomy considers the pelvic fascia to be divided into two parts, the visceral fascia and the mural fascia, with the visceral fascia being the fascia beneath the cellular layer of the serous membrane and surrounding the organs together with the serous membrane, Ercoli et al. Also, between the visceral and mural fascia of the pelvic cavity, there is an extraserosal fascia, which includes the fascial structures around the rectum, uterus, bladder, and Waldeyer’s fascia, and the structure composed of the visceral fascia is more complex in the female pelvis [3]. In the author’s opinion, the proposal of the extraplasma fascia is a more detailed delineation of the extraperitoneal fascial system, and the pelvic extraplasma fascia is a part of the UGF in the pelvis. The UGF from the intermediate mesoderm is highly homologous to the fascia propria of the rectum and the transversalis fascia in terms of origin, but their respective migratory paths during embryonic development are quite different, and the formation of the sacral plane is precisely the result of migration, friction, and fusion between membrane structures. Therefore, when studying membrane anatomy, it is more important to study the migration process, and the extraperitoneal fascial structure of the UGF should be studied as an independent and integral fascial system, and consciously distinguished from the concepts of pelvic visceral fascia and mural fascia. Understanding the anatomy of the UGF has implications for understanding the complex pelvic fascial structures.

The fascial structures surrounding the uterus and the four pairs of ligaments in the parietal uterus play an important role in maintaining the position of the uterus [22, 23]. George Iancu et al. suggested that the broad uterine ligament is part of the intra-pelvic fascia that connects the uterus and upper end of the vagina to the pelvic wall, and mainly functions as a nourisher and maintainer of the anatomical position [24], and Range also confirmed that the broad ligament of the uterus is continued from the branch of the internal iliac artery to the parietal uterus by dissection [25]. This is consistent with our finding that the UGF migrates into the loose connective tissue of the parietal uterus. In the treatment of the pelvic organ prolapse in women, a treatment modality based on strengthening the biomechanical structure of the pelvic fascia has clear implications compared to the serious complications caused by the mesh.

Malignant solid tumors are confined to regions of tissue of the same embryologic origin for a considerable period of time. The boundaries between tissues of different embryologic origins are boundaries for tumors that limit their spread and prevent them from invading adjacent tissues of different embryologic origins. From the point of view of the histo-embryological development of the organ, the key to complete resection of the organ is the precise dissection of the fascial gap between adjacent organs. Total mesometrial resection (TMMR) is a new embryology-based surgical technique for cervical cancer that removes tissue of Müllerian duct origin, removes tissue structures of the same origin in terms of embryonic developmental origins, achieves a very high degree of radicality, preserves the pelvic visceral nerve compared to conventional hysterectomy, and thus reduces the incidence of postoperative complications [26]. However, TMMR neglects to protect the blood vessels within the parietal UGF, and the traditional TMMR surgical plane is within the loose connective tissue of the parietal uterus. According to our anatomical findings, this is the fascial structure encircling the uterus and its vaginal vascular nerves, i.e., the UGF, and if surgery is performed in this plane it is operated inside the UGF, which is prone to cause massive intraoperative bleeding, blurring of the operative field, and increase in operative time. The correct surgical plane should be between the visceral layer of the UGF and the peritoneum, and between the mural layer of the UGF and the transversalis fascia and pelvic wall fascia. Intraoperatively, the borders of the UGF should be found on both sides, and the UGF should be completely freed before carefully dissecting out the important vascular structures, ureters, and nerves and properly protecting them, followed by complete resection of the UGF. Such a surgical idea has some significance in improving the speed of surgery, and operating at the surgical level outside the UGF can significantly reduce the damage to the nerves and blood vessels of the rectum, vagina, and bladder in the UGF, which is helpful to protect the blood supply and function of the rectum and bladder, and to reduce the occurrence of related postoperative complications. Considering the rich lymphatic structure in the UGF, complete resection of the UGF can prevent tumor migration to the greatest extent in the treatment of uterine tumors. The efficacy and safety of gynecological surgery under the guidance of UGF theory lies in the realization of maximum protection of adjacent structures of female internal reproductive organs and minimum trauma to the body, while completely removing surgical lesions, and finally achieving the best treatment effect for patients. The conclusions of this investigation are applicable to patients with no history of pelvic surgery and no anatomical variation, because this may cause changes in the anatomical structure of pelvic UGF, resulting in the inability to clearly distinguish the structure of UGF and its adjacent fascia, which brings limitations to clinical practice. However, with the in-depth understanding and research of the anatomical characteristics of normal UGF, the limitations in clinical practice will be reduced.

The limitations of this investigation are 1. the cadavers were performed on formalin-fixed cadavers, not fresh cadavers, and the findings can be affected by post-mortem degenerative changes.2. because of the limitations of the autopsy technique, it was not possible to remove the entire portion of the UGF in the retroperitoneal, pelvic, and the anterior preperitoneal space from the cadaver intact.3. lack of histologic validation. These deficiencies will be improved in future anatomical studies.

## Conclusion

As in males, the UGF in the extraperitoneal space in females migrates from the perirenal space to the pelvis as the genitourinary organs develop, forming a continuous, integral, and complete fascial structure around them that supports, protects, and nourishes the organs of the genitourinary system. Sorting out its distribution characteristics has some reference value for exploring the metastasis of gynecological tumors, the biomechanical structure of the female pelvis, and the surgical methods of gynecology, colorectal surgery, and hernia surgery.

### Electronic supplementary material

Below is the link to the electronic supplementary material.

Supplementary Material 1

## Data Availability

The datasets generated and analyzed during the current study are available from the corresponding author on reasonable request.

## List of Abbreviations

FNC: Fascial Nomenclature Committee
UGF: Urogenital fascia
TMMR: Total mesometrial resection
